# Three-Dimensional Membranes of Natural Polymer Complex Nanoparticle for Potential Medical Applications

**DOI:** 10.3390/gels9110847

**Published:** 2023-10-26

**Authors:** Mariela Elgegren, Javier Nakamatsu, Betty Galarreta, Suyeon Kim

**Affiliations:** 1 Department of Science, Chemistry Division, Pontificia Universidad Catolica del Peru PUCP, Av. Universitaria 1801, Lima 32, Peru; mariela.elgegren@pucp.edu.pe (M.E.); javier.nakamatsu@pucp.pe (J.N.); bgalarreta@pucp.pe (B.G.); 2 Department of Engineering, Pontificia Universidad Catolica del Peru PUCP, Av. Universitaria 1801, Lima 32, Peru

**Keywords:** wound dressing, aloe vera, acemannan, 3-dimensional membrane, cytotoxicity, lysozyme

## Abstract

Skin wound healing is a complex biological process of tissue regeneration in which the wound dressing is crucial for rapid healing; it must protect the wound keep an adequate level of moisture and prevent infections. Alginate (AL), a polysaccharide from brown algae, has been extensively studied for wound treatment, and aloe vera gels (AVGs) have also been used in the treatment of skin. The AVG main bioactive polysaccharide was combined with AL for the preparation of membranes. Two-dimensional membranes were prepared by casting and, for comparison, transparent nanoparticle 3D membranes were produced by high-intensity ultrasonication followed by ionotropic crosslinking. The effects of the amount of AVG, ionotropic gelation, and the structure (2D or 3D) of the AL-AVG membranes were compared. Scanning electron microscopy (SEM) showed higher surface roughness on 3D membranes. Three-dimensional membranes showed a higher swelling ratio, and swelling increased with AVG content and decreased with higher calcium concentration and longer gelation times. The degradation of the membranes was evaluated with and without a lysozyme at pH 5.5, 7.5, and 8.5, to simulate different skin conditions; the results evidence that pH had a higher effect than the enzyme. The cytotoxicity of the membranes was evaluated with ATCC CCL 163 and ATCC CCL 81 cells, and an excellent biocompatibility of both cell types (>90% of cell viability after 48 h incubation) was observed for all AL-AVG membranes.

## 1. Introduction

The skin is the largest organ protecting the body from external factors such as dehydration, bacteria, chemicals, and heat/cold (temperature). When the skin is damaged by burns, diseases, scratches, or surgeries, its structure and fundamental functions are interrupted by tissue damages [1,2]. Wound healing is a complex biological process of regeneration and restoration of damaged tissues that involves four typical phases, namely, hemostasis, inflammation, proliferation, and remodeling, all of which involve many types of cells, cytokines, and the extracellular matrix (ECM) [3]. Wounds are divided into two types: acute and chronic. Acute wounds are described as a sudden injury to the skin leading to predictable tissue repairing and healing times. On the other hand, chronic wounds are mainly caused by chronic diseases, such as diabetes, cardiovascular and cerebrovascular diseases, hypoxia, cancer, and immunosuppression; this type of wound takes longer to recover and has a difficult healing process [4]. Ideal wound dressings need to accomplish multiple goals, i.e., to have no toxic components leaching into the wound, no foreign body release (for example, non-biodegradable materials such as fibers). Also important is that the wound dressing forms a water-resistant seal to the peri-wound skin that can be removed without causing tissue trauma while maintaining the wound and peri-skin in an optimum state of hydration, and protecting the wound bed and peri-wound skin from damaging exudates and excessive moisture [5]. Antimicrobial and antioxidant activities are also critical in a wound dressing since open wounds are susceptible to persistent infections due to an undesirable pH increase from weakly acidic to higher than 7 (optimum for microorganism growth) and oxidative cell damage caused by excessive release of reactive oxygen species [1,3,4,6]. Wound dressings from natural polymers designed to target the different stages of the pathophysiology of acute and chronic wounds have become an active research topic.

Among many potential natural polymers, alginate has been extensively investigated and applied to the preparation of biomaterials due to its biocompatibility, biodegradability, antimicrobial, non-toxicity, antitumor, antioxidant, immunoregulatory, anti-inflammatory, neuroprotective, hypolipidemic, antihypertensive, and hypoglycemic properties [7,8,9]. In addition to its bioactivities, the ease of ionotropic crosslinking (usually with calcium ions) of the soluble sodium salt of alginic acid to form a hydrogel is also an advantage. For wound dressings, the formation of the alginate hydrogel is advantageous since its structure is similar to the extracellular matrices in tissues and can encourage epidermis regeneration. Moreover, it provides a moist wound environment, which results in the acceleration of wound healing and, at the same time, absorbs the ‘free’ fluid on the wound surface, thus maximizing the wound healing process [10,11].

In this research work, biodegradable membranes for wound dressings were prepared from alginate and aloe vera gel (AVG) as a bioactive component. Aloe vera (AV) is one of the oldest medicinal plants known for its therapeutic activity. AV can store a substantial amount of water in its inner tissue (more than 99% of its total mass) [12,13,14,15]. The interior of the leaves is made of two main components: the aloe gel and a yellow latex (aloe juice). The aloe gel is the commonly used part for food, and as cosmetic and pharmaceutical ingredients. More than 75 biologically active components have been found in the gel: anthraquinones have been identified as being responsible for its various biological activities, such as astringency, hemostatics, antidiabetic, antiulcer, antibacterial, anti-inflammatory, antioxidant, and anticancer activities, and also for its healing efficiency concerning radiation injuries, burns, and general wounds [12,16,17]. Although AV has a long history of clinical use to promote the healing of cutaneous wounds with rare adverse effects, the healing mechanism is not fully understood. Teplicki et al. [18] observed significant stimulatory and protective effects of AVG on the proliferation, migration, and viability of cells related to wound healing and skin regeneration (normal human primary skin fibroblasts and keratinocytes) [18,19]. Hekmatpou et al. [11] reviewed the clinical effects of AV applied to treat diverse wounds such as burns, postoperative, cracked nipples, genital herpes, psoriasis, and chronic wounds, including pressure ulcers, and concluded that AV is very effective at accelerating wound healing for all wound types, with high economic impact and low-risk side effects. First- and second-degree burn wounds improved significantly when AV dressings were applied alone or combined with antibacterial creams [11].

## 2. Results and Discussion

### 2.1. Characterization of the AVG and Acemannan Isolated from AVG

**Figure 1:**
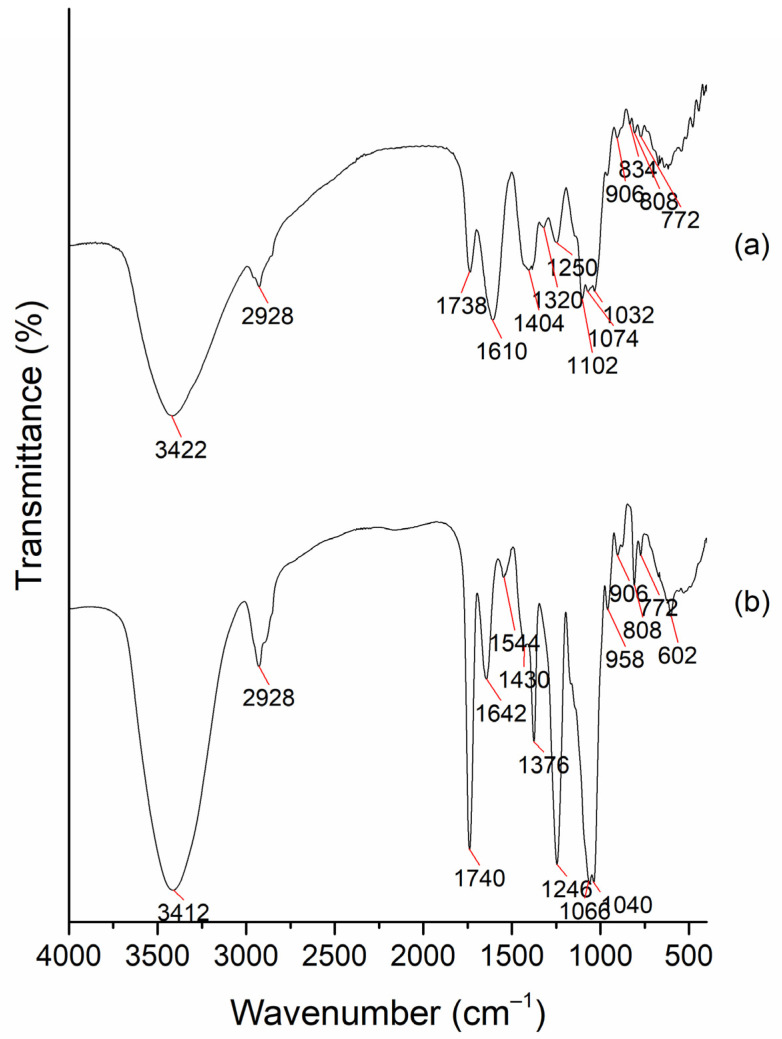
FT-IR of (**a**) lyophilized fresh AVG and (**b**) bioactive polysaccharide extracted from pasteurized AVG (KBr discs).

The chemical composition was analyzed using the FT-IR transmission spectrum shown in Figure 1. The strong band at 3422 cm^−1^ corresponds to a vibration of the -OH group, and the peak at 2928 cm^−1^ corresponds to the stretching vibrations of the C-H aliphatic bonds. Both bands are characteristic of mono- and polysaccharides in general. The weak signal at 1738 cm^−1^ corresponds to C=O groups, and the bands at 1610 and 1404 cm^−1^ are due to asymmetric and symmetric stretching of C-O bonds (from the ester groups, COO^−^). Additionally, it can be seen that the peak at 1032 cm^−1^ evidences the presence of glycosidic bonds, and the one at 808 cm^−1^ is associated with a mannose unit, which is a representative component of acemannan (a main bioactive polysaccharide in aloe gel, as reported in the literature) [20,21]. Similar spectra were found in previous work reported by Pereira et al. [22,23]. The FT-IR spectrum of the major bioactive polysaccharide extracted from pasteurized AVG presented in Figure 1b is similar to that reported by Chokboribal et al. [24] for acemannan polysaccharide. This spectrum is different from the one obtained from the freeze-dried fresh gel shown in Figure 1a. In the spectral region between 900 and 1200 cm^−1^, the intense absorption bands due to the C–O–C bonds’ stretching vibrations can be seen. They are called the saccharide bands; they are sensitive to the conformational changes in the molecules and are directly related to the crystal and amorphous phase in the samples [25]. Peaks at 808 cm^−1^ and 1040 cm^−1^ are due to the glycosidic bonds and mannose units, respectively. Other strong and sharp absorption bands are observed at 1740 cm^−1^ and 1246 cm^−1^, which correspond to the C=O group and the C-O-C stretching, respectively, and are related to the acetyl groups in acemannan [26].

**Figure 2:**
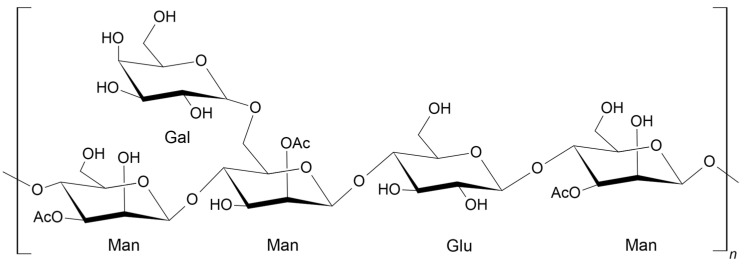
Chemical structure of acemannan.

The polysaccharides in aloe vera are responsible for the physical and biological activities of its gel. The major compositions of these polysaccharides vary depending on growth conditions, such as geographical location, seasonal changes, and the extraction and processing of the parenchyma tissue [13]. Acemannan (acetylated glucomannan) has been identified as the primary polysaccharide in aloe vera gels. Additionally, arabinan, galactan, arabinorhamnogalactan, galactogalacturan, glucogalactomannan, galactoglucoarabinomannan, and other glucuronic acid-containing polysaccharides have been isolated from the inner leaf gel [13]. Among all this diversity of sugars, acemannan is the important one in terms of its biological activity, and has been extensively studied for its pharmacological and biological applications in medical and other industrial fields [27,28]. Acemannan has a backbone composed of β-(1→4)-D-mannosyl units acetylated at the C-2 or C-3 positions, typically with one acetyl group per residue. The backbone also has some galactoses as side groups, which are attached at C-6, as shown in Figure 2 [13].

In this work, the molecular weights (MWs) of polymers of the fresh AVG extract and major bioactive polysaccharide (acemannan) isolated after pasteurization and incubation with ethanol were analyzed using GPC. The results are presented in Table 1. In general, polysaccharide molecules had Mw ranging from around 30~40 kDa up to 1000 kDa. For fresh AVG, macromolecules with a weight-average molecular weight (Mw) of 45 kDa were observed, while the Mw of acemannan was around 22 kDa, possibly due to some degradation during the pasteurization process. These results are similar to the study by Minjares et al. [29], where acemannan was reported to have a molecular weight of 49 kDa that, after pasteurization, diminished to values between 23 to 26 kDa.

**Figure 3:**
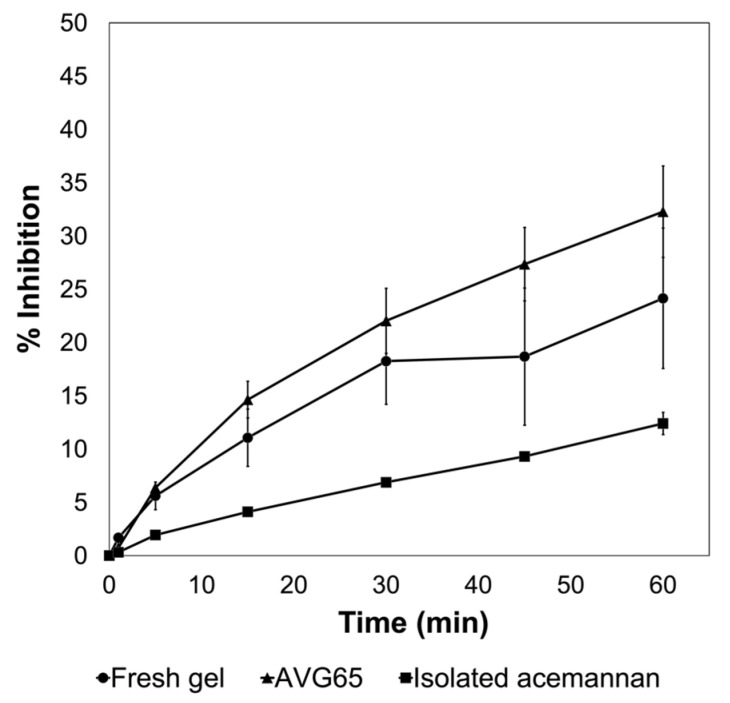
Scavenging inhibition rate of lyophilized AVG extracted from fresh gel, pasteurized at 65 °C gel (AVG65) and acemannan for 60 min.

In addition to polysaccharides, AVG is composed of a variety of other substances, such as polyphenolic compounds. Sumi et al. [16] identified phenolic acid molecules using high-performance liquid chromatography, including catechin hydrate, caffeic acid, ferulic acid, ellagic acid, and quercetin, among others, which are responsible for the gel’s antioxidant activity [16]. Flavonoids are susceptible to oxidation, depending on the pH, water activity, radiation, oxygen, metals, antioxidants, temperature, enzyme activity, storage conditions, and time [17,30]. Figure 3 displays the antioxidant activity of three different samples of lyophilized AVG. The sample with the highest percent of inhibition was the gel previously pasteurized at 65 °C for 15 min (AVG65). After 1 h, the inhibition rate reached 30%. This result is similar to what was reported by Rodriguez et al. [31], who measured the antioxidant activity of aloe vera gel after pasteurization, which might correlate to some improvement in the medicinal properties of the plant. Acemannan isolated from the gel evidenced lower antioxidant activity (only 10% of inhibition after 1 h) when compared to the fresh and pasteurized gels. This result might be due to the purification process during dialysis with a 12–14 kDa cut-off membrane, possibly removing small molecules like phenolic acids and flavonoids, which are mainly responsible for the antioxidant activity.

### 2.2. Effect of Crosslinking on the Swelling Behavior of the 2D and 3D AL Films

**Figure 4:**
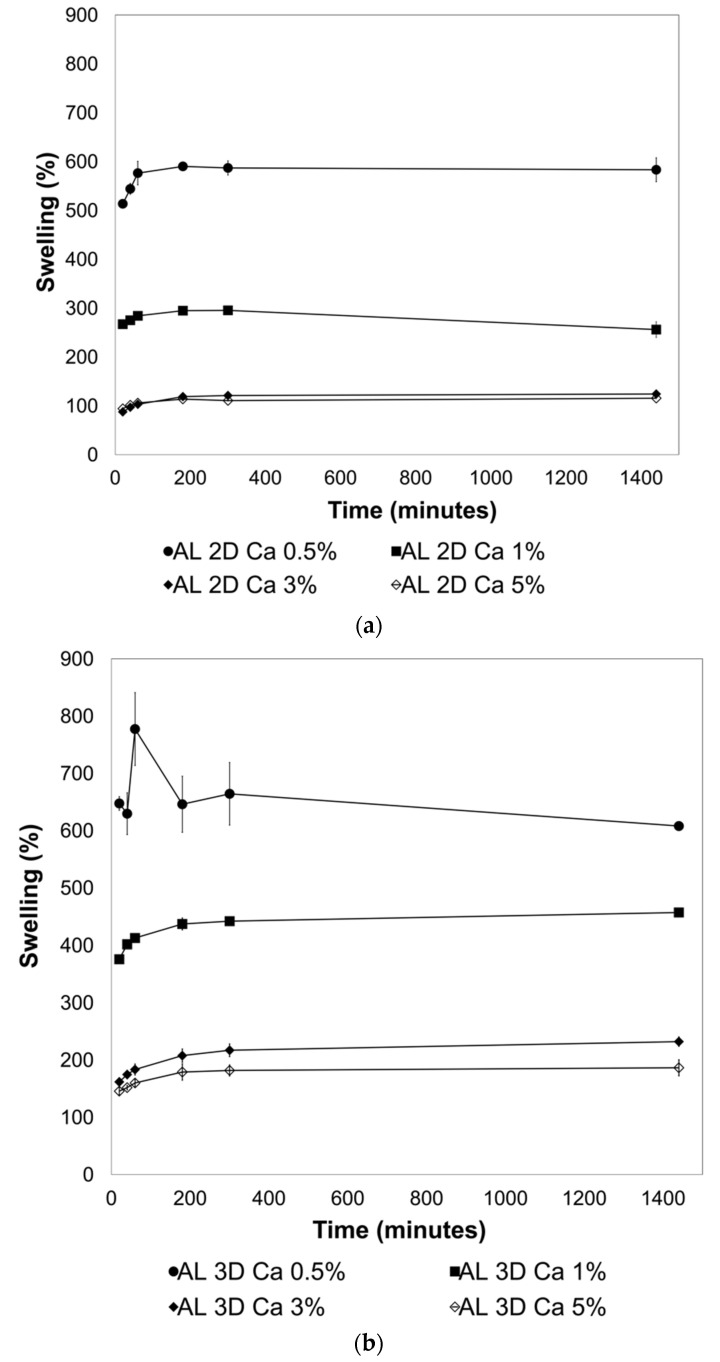
Swelling behavior of the (**a**) AL 2D films and (**b**) AL 3D films crosslinked with different concentrations of CaCl_2_ solution.

AL nanoparticles had an average size of 1014.5 ± 22.6 nm and a polydispersity of 0.57 ± 0.03. As expected, the zeta potential was −29.96 ± 0.26, since AL is an anionic polyelectrolyte. Zeta potential is vital to identifying the surface electric characteristics of particles, and it serves as an indicator of emulsion stability; a high absolute value means a high stability [32]. When AL is combined with AVG, the size and zeta potential changed. The size of the particles increased to 2368 ± 673.8 nm, more than twice the value of AL nanoparticles, with a similar polydispersity (0.50 ± 0.1). A lower value of the zeta potential was obtained for the AL-AVG emulsions: −23.11 ± 2.1. Similar results were presented by Basha et al. [28], who also showed a lower zeta potential when AVG was mixed with AL. The capacity of alginate to form hydrogels by ionic crosslinking in the presence of multivalent ions is well known, and has been used in a range of applications. CaCl_2_ is frequently used for ionic crosslinking of alginate, inducing a sol/gel transition under mild gelling conditions [33,34]. The concentration of Ca^2+^ ions, as well as the crosslinking time, influence the crosslinking degree [35]. When the crosslinking is fully achieved, the hydrogel becomes more rigid, its Young modulus increases, and a lower elongation capacity affects its tensile strength [36]. The degree of crosslinking not only affects the mechanical behavior of the film, but also its water resistance and swelling behavior. To evaluate the effect of CaCl_2_ in the crosslinking of the 2D and 3D AL and AL + AVG films on their swelling behavior, solutions with different concentrations of CaCl_2_ were used: 0.5%, 1%, 3%, and 5% (*w*/*w*). The results are shown in Figure 4. As expected, AL films crosslinked with the lowest concentration of CaCl_2_ (0.5%) resulted in a lightly crosslinked network of chains, exhibiting the highest swelling capacity (approximately 600% by weight) in both 2D and 3D films. At higher calcium concentrations (over 3%), a lower level of swelling was observed: around 100% for 2D and 150–200% for 3D films, respectively. A higher crosslinking density produced by a high concentration of CaCl_2_ limits the water absorption of the film, consequently reducing its swelling capacity [37]. In addition to the effect of CaCl_2_ concentration, the structural features of the film were also compared. As can be seen in Figure 4a,b, a higher swelling efficiency was observed on the 3D structured films for all CaCl_2_ concentration ranges. This may be attributed to an increase in the surface area of 3D films.

### 2.3. Thickness and Transparency of AL-AVG Membranes

The thickness and transparency of the 2D and 3D AL films, prepared with different concentrations of AVG, were measured both before and after crosslinking performed with CaCl_2_ 1% solution for 5 min, and the obtained results are shown in Table 2. Thickness and transparency are important characteristics of the wound dressings as they affect the adhesive efficiency of the wound site and facilitate monitoring the wound healing process, respectively [38]. AL 2D films with a low ratio of AVG (AL-AVG 10) showed a higher thickness compared to those with higher ratios. Among the 2D films with 15%, 20%, and 25% AVG, there was no noticeable difference in thickness before crosslinking. After Ca-crosslinking, film thickness slightly decreases with higher AVG content. The thickness of the films is primarily determined by the AL polymer content because AVG is mainly composed of water (accounting for more than 99% of its total weight), which has minimal impact on film thickness after drying. Total water content of fresh gel was determined to be 99.32% *±* 0.08% by weight, a value that is similar to previous reports [13,14,15].

All the films increased their transparency after crosslinking with CaCl_2_. AL films with 15% AVG content showed the highest transparency values both before and after crosslinking, i.e., 1.98 and 3.03, respectively.

The AL-AVG 3D films prepared with different AVG contents showed a decrease in thickness with an increase in AVG concentration before calcium crosslinking (Table 3). The decrease in the film thickness with higher AVG content was also observed in AL-AVG 3D films after crosslinking, similar to the trend exhibited by the AL-AVG 2D films. Compared to 2D films, 3D films showed higher values of transparency (calculated with Equation (3)). Regarding transparency, there was a slight increase with higher AVG content, in agreement with the results reported by Pereira et al. [39]. Additionally, all films exhibited an increase in transparency after crosslinking with CaCl_2_, as observed in AL-AVG 2D films due to the decrease in thickness. Transparency of a wound dressing plays an important role in reducing the risk of secondary trauma and bacterial exposure that can occur during dressing removal by enabling close and rapid visual monitoring of the wound site without removing the dressing [40].

### 2.4. Morphology of AL-AVG Membranes

**Figure 5:**
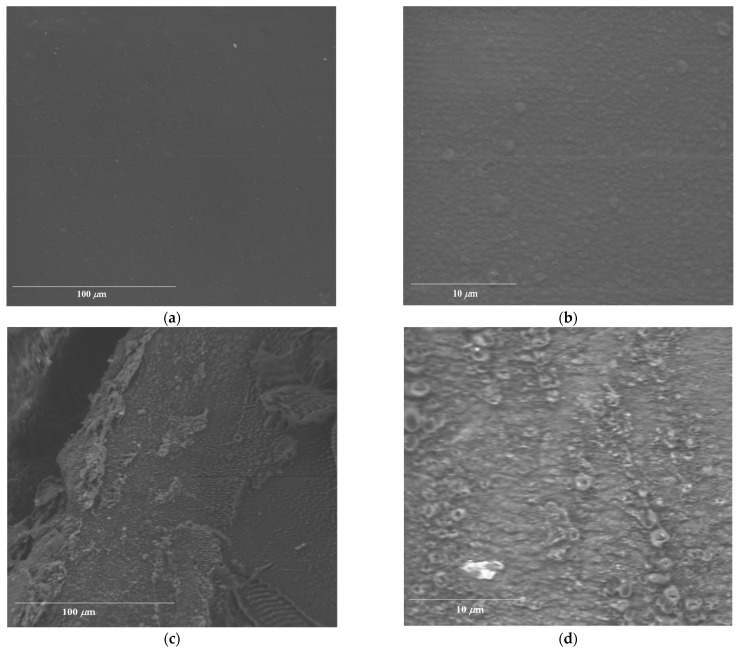
SEM images of films: (**a**) AL-AVG10 2D at 1300× magnification; (**b**) AL-AVG10 2D at 8500× magnification; (**c**) AL-AVG10 3D at 1300× magnification; and (**d**) AL-AVG10 3D at 8500× magnification.

The comparison of the surfaces of the AL-AVG10 2D and 3D films was conducted using a scanning electron microscope (SEM), as can be seen in Figure 5. Figure 5a,b show the surface of the AL-AVG10 2D film with two different magnifications: 1300× and 8500×. In the high-magnification image of the AL-AVG 2D film (Figure 5b), a few particles can be observed, which are probably aggregates or large pieces of the AVG. Even after filtration of the gel extracts, it was difficult to remove all the gel particles, a finding consistent with previous reports by Pereira et al. [34]. Figure 5c,d present the surface morphology of the AL-AVG10 3D films at two different magnifications. The images show a significant difference in surface roughness between the AL-AVG 2D and AL-AVG 3D films, with higher roughness in 3D membranes compared to the 2D films.

### 2.5. Swelling of AL-AVG Membranes

**Figure 6:**
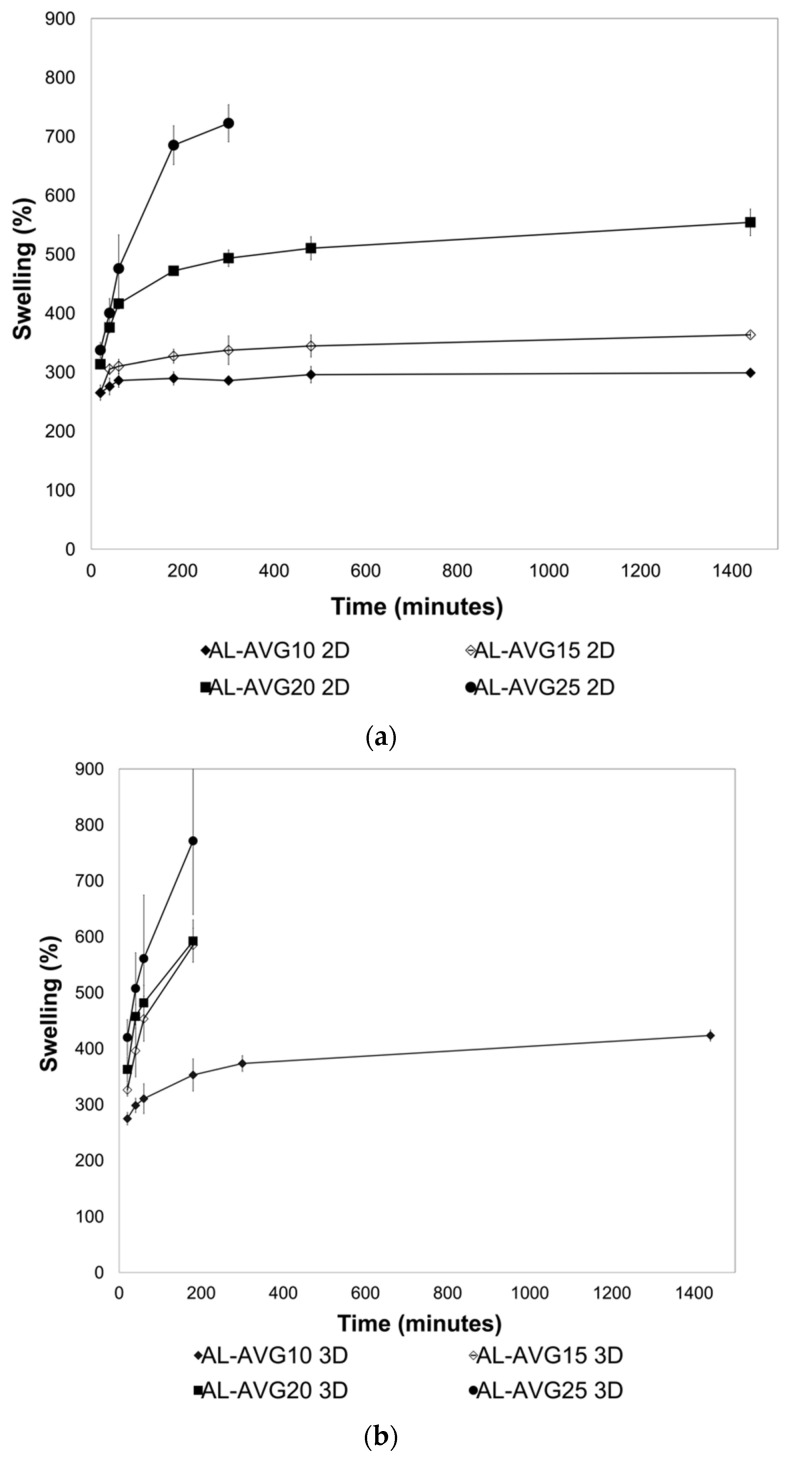
Swelling of the AL-AVG (**a**) 2D and (**b**) 3D films in acetate buffer (pH 5.5, 37.5 °C).

AL dressings can help in wound healing by maintaining constant moisture, absorbing excess exudates from the wound, and reducing local pain through a wound cooling effect [41]. AL provides a moist environment for the wound, stimulating epidermis regeneration. At the same time, AL films have good permeability to water vapor, carbon dioxide, and oxygen, thus protecting the wound from bacterial infections. However, they possess lower swelling capacity than AL hydrogels (which absorb 20 times their weight). Therefore, films are considered less effective for wounds with excessive exudates [9]. In the swelling experiments, the hydration of the polar functional groups in AL and AV chains is responsible for the weight gain of the films. [42]. The swelling behavior of the AL-AVG 2D and 3D films was monitored for 24 h. All tested samples were prepared by crosslinking with a 1% CaCl_2_ solution. The results shown in Figure 6a,b indicate that the amount of AVG incorporated into the AL films is decisive in the swelling capacity, with a higher amount of AVG in the AL films resulting in a greater rate of swelling. The AL-AVG25 2D film (highest AVG ratio) could only be measured for 4 h of swelling since it broke into small pieces that could not be recovered after incubation in the pH 5.5 buffer solution. All the 3D AL films, with exception of the sample prepared with 10% AVG (lowest ratio), showed rapid degradation, possibly due to an increased surface area and an increased capacity of water absorption in these films.

When higher concentrations of AVG were incorporated into the films, higher swelling values were obtained, ranging between 250% and 750% of the original weight of the films. The components of AVG have a high hydrophilic character that contributes to the retention of the aqueous buffer solution. It is important to emphasize that in the cases of AL-AVG25 2D, AL-AVG15 3D, AL-AVG20 3D, and AL-AVG25 3D, the test could not be completed because the swollen films could not be weighed since the membranes broke into pieces. The results in Figure 6a,b show the swelling rate of the AL-AVG films, which depend on the concentration of AVG.

### 2.6. Degradation of AL-AVG Membranes

Considering that the AL 3D films prepared with 15%, 20%, and 25% AVG lost their integrity in the swelling test, the degradation test was carried out only with the AL-AVG10 3D film in various pH conditions: acetate buffer at pH 5.5 and pH 7.5, and ammonium buffer at pH 8.5. Furthermore, the presence of lysozyme in the buffer media was also analyzed.

**Figure 7:**
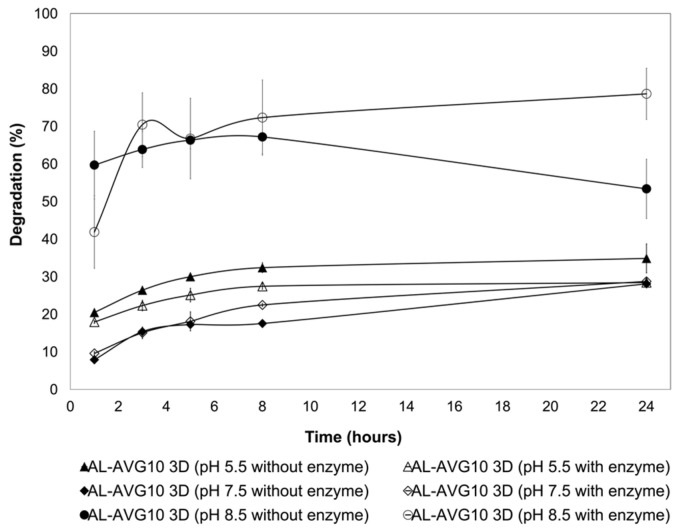
Degradation of AL-AVG10 3D film crosslinked with CaCl_2_ 1% in different media at 37.5 °C.

Lysozyme (*N*-acetylmuramic acid hydrolase E.C. 3.2.1.17) works on the hydrolysis of the β-(1→4)-glycosidic bonds between *N*-acetylmuramic acid (NAM) and *N*-acetylglucosamide (NAG) units in the polysaccharide backbone of the peptidoglycans in the cell wall of Gram-positive bacteria [43]. It is an antiseptic and antimicrobial enzyme found in many mucosal secretions, such as tears, saliva, and mucus, as well as in tissues of animals and plants. It plays an important role in innate immunity, and its critical function in biomedical applications is protection against bacteria, viruses, and fungi [43,44]. Lysozyme derived from diverse sources (like papaya, eggs, and safflower) has been previously used to study skin wound treatments, bone tissue engineering, and the formation of biofilms, showing satisfactory medical effects as well as protection from bacteria like E. Coli and Staphylococcus aureus [6,45]. Biodegradable polymers used as biomaterials can be degraded under physiological conditions (hydrolysis) or by biological systems (enzymatic degradation) in in vivo conditions. In the degradation test, diverse conditions were considered to monitor the degradation behavior of the AL films. In the first place, three buffer solutions were prepared to simulate the different skin conditions depending on the type of wound: slightly acidic (pH 5.5) for skin in healthy normal condition, pH 7.5 for skin with an acute wound, and a more basic pH (8.5) for skin with severe chronic wounds. To complement this, the presence of the lysozyme was also evaluated and compared with the degradation behavior of the films. The results in Figure 7 illustrate the dependence of the degradation of the films on both pH and the presence of the enzyme.

Under basic conditions (pH 8.5), the mass loss of the AL-AVG10 3D membrane was higher and faster than under pH 5.5 and 7.5 conditions. At this basic pH, the higher solubility of alginate (an anionic polyelectrolyte) is probably responsible for the degradation of the films. The effect of the enzyme on the degradation of the films was not remarkable at pH 5.5 and 7.5; however, a significant increase in the degradation of films was observed at pH 8.5 after 5 h incubation. Enzyme activity is affected by reaction conditions, especially pH and temperature. Lysozyme presents maximum activity at pH 5 and it decreases under acidic conditions (lower than pH 3.8) and alkaline conditions [46]. Considering the lysozyme activity, the degradation behavior of AL films was more influenced by the pH than by enzymatic hydrolysis. The results evidence that the AL-AVG10 3D films are suitable for chronic wound dressings since they degrade rapidly and are able to release any incorporated active components to the wound site as quickly as possible. The lowest film degradation was observed under neutral conditions (pH 7.5, acute wound simulated conditions), i.e., less than 25% degradation even after 24 h.

### 2.7. Surface Wettability of AL-AVG Membranes

**Figure 8:**
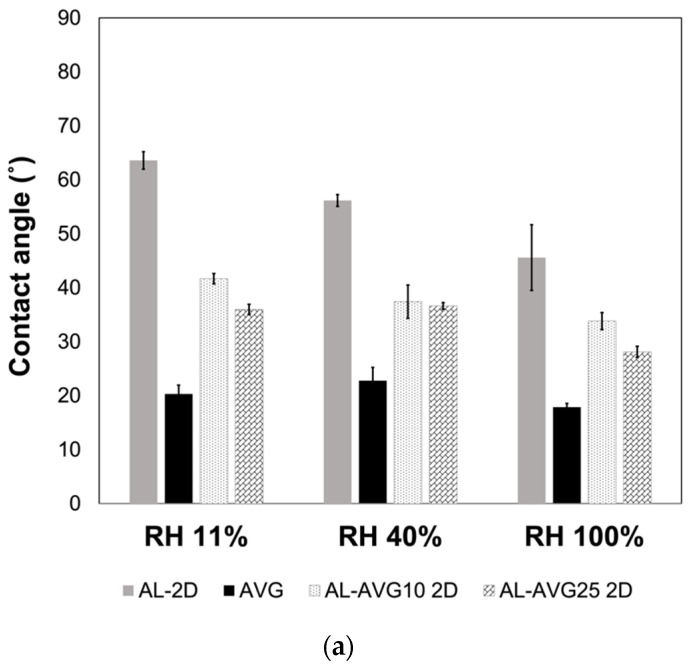

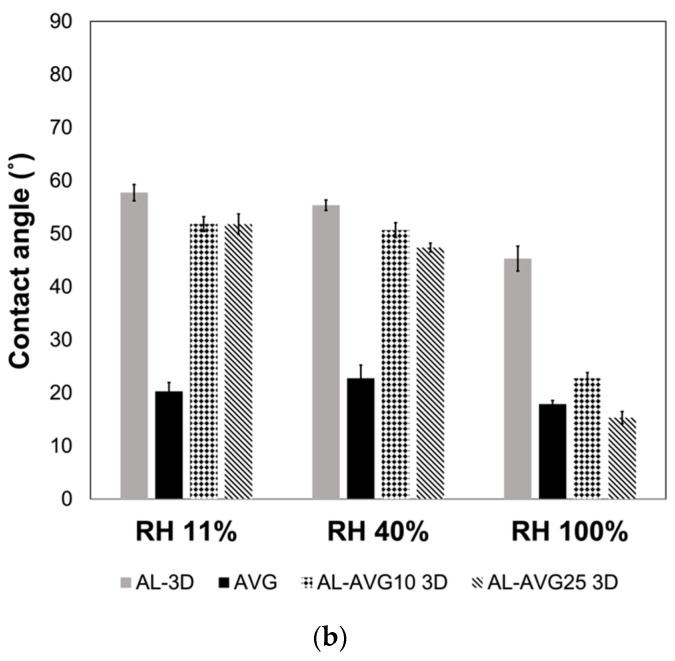
Contact angles of a drop of water on AL, AVG, AL-AVG10, and AL-AVG25 (**a**) 2D and (**b**) 3D films at different relative humidity (RH).

The hydrophilicity of the AL and AL-AVG films was evaluated through contact angle measurements of a water droplet in different humidity environments. As can be seen in Figure 8a,b, all the films showed contact angles lower than 90°, indicating their hydrophilic nature. For wound healing materials, hydrophilicity in dressings is important since it allows for the absorption and evaporation of wound exudates, helping to minimize bacterial contamination. AL is inherently hydrophilic, making it suitable for use in post-traumatic wounds or those with exudate [8,34,36,41]. In both 2D and 3D structured films, those made of AL displayed the highest contact angles (less hydrophilic), while films containing only AVG were more hydrophilic, with the lowest contact angles (around 20°). As expected, a higher content of AVG in the films increased their hydrophilicity, leading to lower contact angles [20]. The ambient relative humidity under which the films were stored had a small effect on the hydrophilicity of the films, with films stored at higher humidity conditions showing lower contact angles.

### 2.8. Cytotoxicity of AL-AVG Membranes

**Figure 9:**
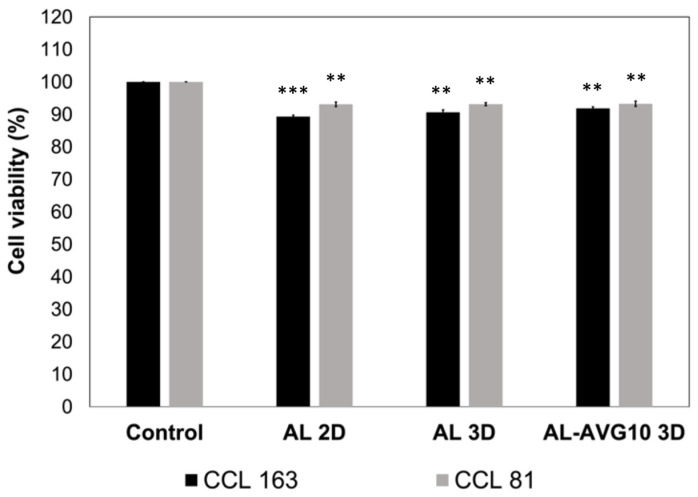
Cytotoxicity of 2D AL, 3D AL, and 3D AL-AVG films determined by cell viability of CCL 163 and CCL 81 cells over 48 h. Viability was obtained by comparison with the negative control (no treatment). Data are presented as mean ± SD (*n* = 3). Statistically significant differences were achieved by comparing control and AL membranes. *** *p* < 0.001; ** *p* < 0.01. The degree of freedom is 2.

It has been reported that both AL and polymers in AV have good biocompatibility [33,34,41,47]. An MTT assay protocol was followed with CCL 163 and CCL 81 cells to evaluate the biocompatibility of the AL 2D, AL 3D, and AL-AVG 3D films. After 48 h of incubation, all the tested samples showed over 90% cell viability and a high cell survival rate with both CCL 163 and CCL 81 cell types (Figure 9). Notably, in all evaluated samples, slightly higher cell viability was observed with CCL 81 Vero cells compared to CCL 163 fibroblast cells. These results are in agreement with a study conducted by Carvalho et al. [47], which showed that AV exhibited almost no reduction in human pulp fibroblast (FP6) cells over 72 h, confirming its excellent biocompatibility.

## 3. Conclusions

Biodegradable wound dressings can be produced with readily available natural materials that are already known/used for wounds on skin, namely, alginate and aloe vera gels. In this study, the membrane structure was varied, 2D vs. 3D, to determine how this would impact the wound exudate absorption and degradation behavior of the membrane. Three-dimensional membranes have the advantage of being able to easily incorporate other bioactive compounds, especially those with low water solubility. Three-dimensional membranes were produced from an AL and AVG nanoemulsion formed by ultrasonication. The level of crosslinking (controlled by CaCl_2_ concentration and time) affected the physical and chemical properties of AL-AVG membranes. AL-AVG 3D films showed high transparency, which is advantageous for visible wound monitoring without removing the dressing. The AVG-incorporated AL films are hydrophilic and are suitable for skin protection applications. The amount of AVG incorporated into AL membranes was crucial for swelling, with higher swelling occurring with increased AVG content. Due to their high surface area, 3D structured membranes presented an increased swelling ratio compared to 2D membranes. A higher swelling capacity allows membranes to be used in wounds with high exudate levels, while a lower swelling capacity allows them to be used in the final stages of wound healing. AL-AVG 3D films also displayed faster weight loss at a basic pH of 8.5, simulating skin with severe chronic wounds. This suggests that it may be a suitable dressing for chronic wounds and can be used for the rapid release of bioactive compounds to the wound site to accelerate the healing process. This can be demonstrated through in vivo wound healing and epithelial cell regeneration experiments. The membranes showed very low cytotoxicity, proving their safe use for wound treatment.

## 4. Materials and Methods

### 4.1. Materials

Sodium alginate (AL) from brown algae was purchased from Sigma-Aldrich (St. Louis, MO, USA). Fresh aloe vera (Aloe barbadensis) leaves were obtained from a local herbalist (Lima, Peru) and were processed the same day they were acquired. Cellulose dialysis membrane with a pore size of 12–14 kDa was purchased from Sigma-Aldrich. Lysozyme (EC 3.2.1.17) from chicken egg white was obtained from Sigma-Aldrich and used to evaluate the enzymatic degradation of samples. Non-ionic surfactant poloxamer 407 and other chemicals were also purchased from Sigma-Aldrich and used without any further treatments.

### 4.2. Aloe Vera Gel (AVG) Preparation and Characterization

#### 4.2.1. AVG Extraction

The extraction was performed following the process proposed by Silva et al. [48]. Firstly, fresh aloe vera leaves were washed with distilled water to remove any dirt on the surface. Then, the spines and the entire green cuticle external layers were removed. The internal gel was then washed with plenty of distilled water to remove the yellow exudate. The gel was cut into small pieces and ground with an immersion blender. The ground AVG was centrifuged for 10 min at 3500 rpm and filtered using a pressure metal filter. The prepared AVG was then refrigerated at 4 °C for further use.

#### 4.2.2. Isolation of Bioactive Polysaccharides of AVG

Isolation of the main bioactive polysaccharides in AVG was carried out following a technique proposed by Salah et al. [49]. The ground and filtered AVG was pasteurized by heating it at 65 °C for 15 min, and then it was filtered again. Ethanol was added to the pasteurized AVG at a ratio of 1 to 3 (*v*/*v*) and left for 12 h at room temperature. The insoluble solids in the ethanol were recovered by centrifugation at 3000 rpm for 20 min and then dissolved in distilled water at a concentration of 1.5 mg/mL. The pasteurized AVG was centrifuged again to collect the soluble portion, which was placed in a dialysis cellulose membrane (pore size of 12–14 kDa) in ultrapure water and finally lyophilized.

#### 4.2.3. Evaluation of Total Water Content of AVG

After removing the exudate from the initial gel (see Section 4.2.1 AVG extraction), it was cut into cubes of approximately 1 cm. The excess water on the surface of the gel was dried with filter paper, and the cubic gel was weighed (*w_gel_*). The gel was dried at 50 °C until a constant mass (*w_dry gel_*) was reached, and the water content of the aloe vera gel was calculated following Equation (1). This analysis was performed in triplicate, and the average value was determined.

(1) $$Total\;water\;content\;\left(\%\right)=\frac{w_{gel}-w_{dry\;gel}}{w_{gel}}\times 100$$

#### 4.2.4. Fourier-Transformed Infrared (FT-IR) Analysis

The chemical characteristics of AVG were studied using a FT-IR spectrophotometer (Perkin Elmer Frontier). After removing the exudate, gel samples were dried at 50 °C and ground together with potassium bromide (KBr) at 1:100 (*w*/*w*), and then pressed to form a KBr pellet. The transmission spectrum was obtained between 400 and 4000 cm^−1^, with 4 cm^−1^ resolution and 32 scans. FT-IR analysis was repeated with a lyophilized sample obtained after the isolation of the major polysaccharides of AVG (see Section 4.2.2).

#### 4.2.5. Gel Permeation Chromatography (GPC)

The molecular weight distribution of the soluble polysaccharides was determined following a previous study by Turner et al. [50] with some modifications. An aqueous solution of 0.2 M NaCl was prepared for the mobile phase and then vacuum filtered with a 0.22 μm membrane filter. To prepare the sample, the soluble components in extracted AVG were mixed with the mobile phase at a ratio of 1:1 (*v*/*v*) and filtered with a 0.45 μm syringe filter. Chromatography was performed at a flow rate of 0.5 mL/min at 35 °C in a Viscotek model A6000M (300 *×* 8 mm) column using a GPC system (Malvern Viscotek, Malvern Panalytical, Great Malvern, UK). Four pullulan standards (PSS Polymer Standards Service, Mainz, Germany) with molecular weights ranging between 10 kDa and 800 kDa were used as molecular weight references (at 1 mg/mL concentration). GPC analysis was also performed with the solid compound obtained from the isolation of the major polysaccharide of AVG. A 1 mg/mL solution was prepared with a 0.1 M NaCl mobile phase and then filtered with a 0.45 μm syringe filter. The analysis conditions were the same as detailed above.

#### 4.2.6. Antioxidant Activity Evaluated by ABTS Decoloration Assay

The antioxidant activity was evaluated following the guidelines described by Re et al. [51]. A stock solution of 7 M 2, 2′-azino-bis (3-ethylbenzothiazoline-6-sulphonic acid), ABTS, and 2.45 M potassium persulfate in ultrapure water was prepared and kept in the dark for at least 12 h. The solution was then diluted in ethanol until an absorbance of 0.7 (±0.01) at 734 nm was reached using an UV–Vis spectrophotometer (Thermo Scientific Genesys 50, Waltham, MA, USA). The absorbance of 3 mL of the diluted ABTS in ethanol was measured (*A_control_*) and set as the control. Subsequently, 10 mg of the lyophilized sample of AVG was placed in 3 mL of the diluted ABTS, and the absorbance was measured at 734 nm (*A_sample_*) at different time periods. The inhibition percentage was calculated using Equation (2). The analysis was repeated three times, and the average value was calculated.

(2) $$Inhibition\;\left(\%\right)=\frac{A_{control}-A_{sample}}{A_{control}}\times 100$$

Three lyophilized samples were evaluated: the fresh gel extracted from aloe vera (prior to pasteurization), the AVG extracted using the procedure described in Section 4.2.1, and the major polysaccharide isolated from AVG (Section 4.2.2).

### 4.3. AL-AVG Membrane Preparation

**Figure 10:**
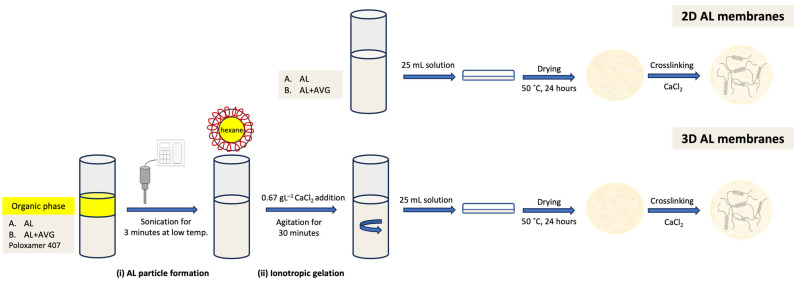
Schematic process for the preparation of the 2D and 3D AL (or AL-AVG) membranes.

Two different AL membranes were prepared: (i) a two-dimensional (2D) membrane prepared from an AL solution and (ii) a three-dimensional (3D) membrane prepared from an AL nanoemulsion. A comparative evaluation was carried out to determine the structural features of each of the AL membranes. All membranes were prepared with an AL solution of 1% (*w*/*v*) using a casting and solvent evaporation technique previously published [52,53,54]. A sonication process was first carried out to obtain the 3D AL membranes. AL or AL-AVG solutions were subjected to high-intensity ultrasonication for 3 min, controlling the temperature with an ice bath (7–10 °C) [31]. Hexane was used as an organic phase to produce the nanoemulsions, and 0.5% poloxamer-407 was used as a stabilizer. After ultrasonication, the ionotropic gelation was achieved by adding a 0.67 g/L CaCl_2_ solution to the resulting emulsion under magnetic agitation for 30 min. In the production of AL-AVG membranes, pasteurized AVG solution was incorporated due to its high antioxidant activity. A schematic process for the 2D and 3D membrane production is presented in Figure 10. The physicochemical analysis of the AL and AL-AVG nanoparticles in the emulsions was carried out by measuring the particle size and polydispersity, and the zeta potential was determined using a Zetasizer Nano series (Malvern Instruments Inc., Worcester, UK). Appropriate dilution of the emulsion of nanoparticles was performed with ultrapure water. All measurements were repeated at least three times, and the average values are presented.

After casting the 2D and 3D AL films, crosslinking with CaCl_2_ was carried out. The optimization of the crosslinking degree was evaluated by varying the concentration of CaCl_2_. Four concentrations were used: 0.5%, 1%, 3%, and 5% (*w*/*v*). After crosslinking, the films were kept in a desiccator at room temperature with relative humidity < 10% until use. The effects of the crosslinking degree on the thickness and swelling (at pH 5.5, 37 °C) of films were evaluated. In addition to varying the crosslinking degree, different mixing ratios of AVG to AL solutions were also examined for their film characteristics. The mixing ratios of AL and AVG are shown in Table 4.

### 4.4. Characterization of the AL-AVG Membranes

#### 4.4.1. Thickness and Optical Transparency of AL-AVG Membranes

The thickness of the dry crosslinked membranes was measured at six different points, including the center, using a thickness gauge (Mitutoyo, Aurora, USA), and the average (*x*) was calculated in micrometers. The optical transparency of the AL-AVG membranes was measured as described by Norajit et al. [55]. The absorbance of the membranes was determined at 600 nm (*A*_600_) using a UV–Vis spectrophotometer (Thermo Scientific Genesys 50). The average of six measurements was obtained (*A*_600_) and the transparency was calculated following Equation (3), where *x* is the membrane thickness:

(3) $$Transparency=\frac{A_{600}}{x}$$

#### 4.4.2. Surface Morphology

The analysis of the film surfaces was carried out using a Delong America LVEM5 multimode electron microscope (SEM). A piece of film was placed on a support with double contact copper tape and analyzed under ultrahigh vacuum conditions with an applied energy of 5 keV. The films evaluated were AL-AVG10 2D and AL-AVG10 3D.

#### 4.4.3. Swelling and Degradation of AL-AVG Membranes

Square pieces of the films (2 × 2 cm) were kept in a desiccator at room temperature (relative humidity < 10%) for 24 h. Each sample was weighed (*w_i_*) and then immersed in a 10 mM sodium acetate buffer pH 5.5 (similar to the pH of healthy skin) at 37.5 °C ± 0.1 °C (body temperature). The films were left in the buffer for 24 h, and the weight increase was monitored after 20 min, 40 min, 60 min, 180 min, 300 min, and 1440 min. To measure swelling and degradation, the film’s surface was lightly dried with kimwipes (*w_h_*). Three pieces of each film were evaluated, and the swelling percentage was calculated using Equation (4).

(4) $$Swelling\;\left(\%\right)=\frac{w_{h}-w_{i}}{w_{i}}\times 100$$

To determine the degradation of the films, after completing the swelling test, they were dried in a desiccator with silica gel at room temperature for 2 days and weighed (*w_d_*). The loss of mass was calculated with respect to the mass of the initial film using Equation (5). The degradation of the films in a wound was simulated using a solution mimicking a wound exudate composed of 0.02 M CaCl_2_, 0.4 M NaCl, 0.08 M tris (hydroxymethyl)aminomethane, and 2% (*w*/*v*) bovine serum albumin [56]. The wound level was controlled by pH, with a simulated normal wound at pH 7.5 and a chronic wound at pH 8.5 (with NH_3_/NH_4_Cl buffer). The tests were carried out for 24 h at three different pH conditions, both with and without 0.1 g/L lysozyme, to verify the enzymatic degradation behavior in terms of biostability.

(5) $$Degradation\;(\%)=\frac{w_{i}-w_{d}}{w_{i}}\times 100$$

#### 4.4.4. Wettability of AL-AVG Membranes

To evaluate the wettability of the 2D and 3D films, they were cast on a piece of flat glass at the same conditions as mentioned in Section 4.2.3. above. Films were completely dried at 50 °C for 24 h and then stored at room temperature in a conditioning case with a relative humidity (RH) of 11%. The wettability of the AL-AVG films was evaluated by measuring the contact angle of a water droplet on the surface using a goniometer (ramé-Hart 250). The contact angle was obtained using the sessile drop technique with a 5 µL droplet of ultrapure water, and 50 measurements were taken at intervals of 0.005 s. The analysis was performed at six different points along the sample. The test was also performed on films that were conditioned at 40% and 100% RH to evaluate the effect of different ambient conditions on the variation in surface wettability of the films.

#### 4.4.5. Cytotoxicity

The cytotoxicity of the AL, AL 3D film, and AL-AVG10 3D film was evaluated following the ISO 10993-5 standard method [57]. The in vitro cytotoxic effects of the samples were tested with two different cell lines, namely, BALB/c 3T3 fibroblast (ATCC CCL 163) and Vero (ATCC CCL 81) cells, using the MTT (3-[4,5-dimethylthiazol-2-yl]-2,5 diphenyl tetrazolium bromide) metabolic reduction method. All the samples were sterilized before the test to avoid microbial contamination in cell culture.

Cells were inoculated into 96-well tissue culture polystyrene (TCPS) plates and incubated at 37 °C in a humidified atmosphere of 5% CO_2_ and 95% air for 24 h to allow cells to attach to the plates. A plate containing each cell line was fixed in situ with trichloroacetic acid to obtain the values at time zero before adding the sample. Then, the plates were stained with MTT and received the corresponding sample. Incubation was carried out for 48 h. The assay ended by removing the culture medium. The culture microplates were washed out with phosphate buffer saline (PBS) to avoid interference from serum proteins in the final formazan (artificial chromogenic dye) dilution. Then, 100 µL of MTT (0.25 mg/mL concentration) was added to an unsupplemented medium, and all the plates were incubated for 4 h under standard conditions. The supernatant was discarded, and 100 mL of dimethyl sulfoxide (DMSO) was added to dissolve the formazan, a product of MTT metabolism. The plates were analyzed in a microplate reader at 500 nm. The culture medium itself was used as a negative control.

### 4.5. Statistical Analysis

Data were statistically analyzed through t-tests using Excel and a *p* value < 0.05 was considered as statistically significant. Data are presented as the mean ± standard deviation with sample number (n).

## Figures and Tables

**Table 1:** Molecular weights and polydispersity of AVG and acemannan from GPC.

	AVG	Acemannan
Mn (kDa)	34.7	3.3
Mw (kDa)	45.3	22.2
Polydispersity (Mw/Mn)	1.31	6.83

**Table 2:** Thickness and transparency of the AL-AVG 2D films before and after crosslinking.

	Before		After	
Film	Thickness (µm)	Transparency	Thickness (µm)	Transparency
AL-AVG10	28.00 ± 1.01	1.78 ± 0.03	27.83 ± 0.71	2.24 ± 0.27
AL-AVG15	25.42 ± 1.06	1.98 ± 0.09	25.17 ± 0.94	3.03 ± 0.22
AL-AVG20	25.92 ± 1.06	1.95 ± 0.11	24.23 ± 0.82	2.57 ± 0.48
AL-AVG25	25.11 ± 0.42	2.02 ± 0.03	23.22 ± 0.92	2.76 ± 0.63

**Table 3:** Thickness and transparency of the AL-AVG 3D films before and after crosslinking.

	Before		After	
Film	Thickness (µm)	Transparency	Thickness (µm)	Transparency
AL-AVG10	30.11 ± 0.84	4.30 ± 0.25	21.39 ± 0.25	5.71 ± 0.62
AL-AVG15	27.92 ± 0.12	3.97 ± 0.33	25.25 ± 2.24	3.60 ± 0.22
AL-AVG20	28.58 ± 1.06	4.53 ± 0.02	20.08 ± 0.59	4.56 ± 0.25
AL-AVG25	21.83 ± 0.47	4.34 ± 0.03	18.42 ± 0.35	4.17 ± 0.03

**Table 4:** Mixing ratios (%) between AL 1% (*w*/*v*) solution and AVG in the preparation of AL membranes.

Sample	AL 1% (% *v*/*v*)	AVG (% *v*/*v*)
AL	100	-
AL-AVG10	90	10
AL-AVG15	85	15
AL-AVG20	80	20
AL-AVG25	75	25

## Data Availability

The data presented in this study are openly available in article.
